# Taxonomy—Dependent Seed Tocochromanol Composition in the Rutaceae Family: Application of Sustainable Approach for Their Extraction

**DOI:** 10.3390/plants15030455

**Published:** 2026-02-02

**Authors:** Danija Lazdiņa, Inga Mišina, Krists Dukurs, Paweł Górnaś

**Affiliations:** Institute of Horticulture, Graudu 1, LV-3701 Dobele, Latvia; danija.lazdina@lathort.lv (D.L.);

**Keywords:** Sichuan pepper, orange, lemon, lime, curry tree, Ruta, tocols, lipophilic antioxidants, bioactive phytochemicals

## Abstract

Several members of the Rutaceae (citrus) family are widely cultivated and processed. Tocopherol (T) synthesis and composition are well-documented, while tocotrienols (T3) in most plant families remain underreported. To amend this, mass screening of Rutaceae species’ seed tocochromanols were analysed. Of the 53 analysed species, seed tocochromanols were tocotrienol-dominated in 22 species, including a majority of species Zanthoxyloideae (*Choisya*, *Dictamnus*, *Melicope*, *Ptelea*, *Skimmia*, *Tetradium*, *Zanthoxylum*) and the Cneoroideae (*Cneorum*) subfamily. Total tocochromanol content ranged from 0.20–25.98 mg 100 g^−1^ dry weight (dw) seeds. The highest tocochromanol content was observed in *Murraya paniculata*, *Ruta graveolens* seeds, the highest tocotrienol (T3) content was observed in *Skimmia anquetilia* and *Dictamnus albus*—19.80 and 19.70 mg 100 g^−1^ dw, respectively. The major tocochromanols in the seeds were γ-T and γ-T3, while others were present in low concentration or absent. Linear discriminant analysis (LDA), principal component analysis (PCA) and non-hierarchal cluster analysis (N-HCA) identified similar tocochromanol content trends in the Rutoideae subfamily species and the *Bergera* and *Murraya* genus, while the Zanthoxyloideae subfamily species’ seed tocochromanol composition was highly variable. The efficient extractability of tocochromanols using sustainable solvent–ethanol is demonstrating suitability of this approach for daily samples screening and bioactive extraction.

## 1. Introduction

There are about 162 genera and over 2085 species in the Rutaceae, commonly called the rue or citrus family. Additionally, several hybrid species have been produced. Recent phylogenetic studies using molecular markers divide the family into six subfamilies [1] (*n*_genera_; *n*_species_ are provided in parentheses): Amyridoideae (3; 42), Aurantioideae (27–28; 206), Cneoroideae (8; 35), Haplophylloideae (1; 66), Rutoideae (5; 20) and Zanthoxyloideae (109; 1700). Most species are trees or shrubs, with some herbaceous exceptions—*Ruta*, *Boenninghausenia* and *Dictamnus*. Several species in the genus are cultivated in large amounts. The *Citrus* genus is most widely known, cultivated, processed, and consumed [2]. Many of the species produce aromatic leaves and fruits [2], and are therefore used as spices—Sichuan pepper (several members of the *Zanthoxylum* genus) [3] and kaffir lime (*Citrus hystrix*) and curry leaves (*Bergera koenigii*) [4]—or perfumery (citrus flowers and peel) [5]. Others’ use is limited due to the presence of alkaloids and photodermatitis-inducing compounds [2].

**Figure 1:**
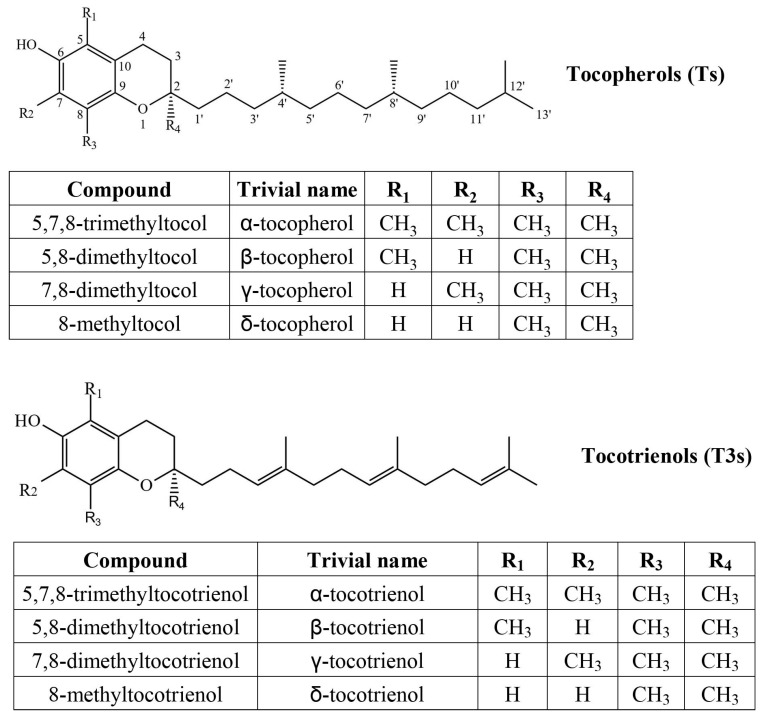
Chemical structures of four tocopherol and four tocotrienol homologues.

Tocochromanols are a group of lipophilic secondary metabolites. Their common features include a polar chromanol ring with varying placement of methyl groups and a hydrophobic isoprenoid tail. The most common representatives are tocopherols (Ts) and tocotrienols (T3s), which have analogous chromanol ring structure and the same tail length, but tocotrienols have three double carbon–carbon bonds in the tail, while in tocopherols the chain is saturated (Figure 1).

Tocopherols and tocotrienols have a common biosynthesis pathway—they are biosynthesised in chloroplasts, the chromanol ring is produced from homogentisate, produced via the shikimate pathway and the same enzymes are responsible for cyclization and methylation of the chromanol ring, but the isoprenoid chains have different precursors. Tocotrienols solely use geranylgeranyl pyrophosphate (GGPP), produced via the methyl erythritol pathway (MEP), as the chain precursor, while tocopherols use phytyl pyrophosphate (PPP), which can be produced from GGPP in MEP or from degraded chlorophyll via the phytol recycling pathway. GGPP or PPP is condensed with HGA by substrate-specific prenyltransferases—homogentisate phytyltransferases preferably use PPP, while GGPP is used by homogentisate geranylgeranyltransferases [6,7]. However, homogentisate phytyltransferases have been observed to use GGPP in transgenic *Arabidopsis* (Brassicaceae family) plants if there is overaccumulation of GGPP and HGA [8].

The primary functions of tocochromanols in plants are considered membrane stabilization and lipid peroxyl radical scavenging [9]. They are also involved in the modulation of gene expression and plant stress signalling, and mutants with modified tocochromanol synthesis have disrupted germination, reduced stress tolerance and modified metabolite accumulation [10]. Tocopherols are more common in plant material—they are found in all parts of the plant. Consequently, tocotrienol content is analysed less often and their presence is likely underreported even if tocopherol synthesis is documented and understood well in the plant. While tocochromanols have been investigated in Rutaceae species’ fruit flesh, seeds and seed oils, most only investigate tocopherols [11,12,13,14,15], and very few reports on tocotrienol contents are available. When tocotrienols are investigated, the main reported tocochromanols in citrus are α-T, followed by γ-T3 and α-T3 [16,17]. Citrus seeds and their seed oils represent the most thoroughly investigated species in the Rutaceae family concerning tocochromanol composition. An overwhelming majority of earlier studies did not include tocotrienol standards in their analytical protocols [13,18,19,20,21]; however, a few investigations that incorporated such standards have consistently detected measurable tocotrienol levels [22,23,24]. These findings underscore the need for a broader and more systematic survey of non-citrus Rutaceae taxa to identify additional—and potentially richer—sources of tocotrienols within the family.

Since the expression of genes related to PPP-producing enzymes (GGPP reductase) and phytol recycling (phytol kinase and phytyl-P kinase) is reduced during citrus fruit maturation, while relative expression of genes related to HGA (tyrosine aminotransferase and 4-hydroxyphenylpyruvate dioxygenase) and GGPP-producing enzymes (GGPP synthase) is increased [7], there is sufficient precedent for tocotrienol research in citrus and other Rutaceae species. For example, in honey and golden pomelo, tocotrienols constitute a significant portion of total tocochromanols throughout fruit maturation, while tocopherol content is reduced [16]. Homogentisate geranylgeranyl transferase activity during fruit maturation has not been studied in depth. It is important to further emphasise that in seeds of both examined pomelo genotypes (*Citrus maxima*), α-T—the sole tocopherol detected—appears to decrease progressively during fruit ripening, whereas the tendency of tocotrienols across maturation remains unclear [16]. By contrast, grape seeds (*Vitis vinifera*) tocopherols dominate early in seed development, followed by a logarithmic accumulation of tocotrienols during the latter half of maturation, ultimately resulting in a tocotrienol-dominated profile [25]. These divergent observations underscore the need for more systematic investigations into temporal changes in tocochromanol composition during seed development, particularly to elucidate how a shared biosynthetic machinery is differentially regulated across species and developmental stages.

Chemotaxonomy is a useful searching tool for plants rich in tocotrienols. Its application is based on the following formula. If the presence of tocotrienols has been reported in plant species “*species 1*” and “*species 2*” belonging to the same family “family 1”, then there is a high probability to find the presence in not yet investigated plant species “*species 3*”, “*species 4*”, “*species n*”, which also belong to the same family “family 1”. Using this approach, a dominance and new sources of tocotrienol were found in the seeds of the Vitaceae species [26]. However, such aspects as the distribution of relevant secondary metabolites and significant effects of factors affecting their production, such as ontogenetic, diurnal, and seasonal variation, should be taken into account. Therefore, despite preliminary findings that indicate that certain classes of secondary metabolites might have chemotaxonomic utility at lower taxonomic levels, a cautionary note must be added [27]. Therefore, the aim of the present research paper is to provide a detailed accounting of tocochromanols in Rutaceae species’ seeds and tocochromanol profile relation to the taxonomic classification of the species as well as the establishment of a rapid, robust, and reproducible extraction protocol for these lipophilic bioactive compounds, which is both effective and health-safe.

## 2. Results and Discussion

### 2.1. Saponification and UAEE Recovery and Measurement Repeatability

**Figure 2:**
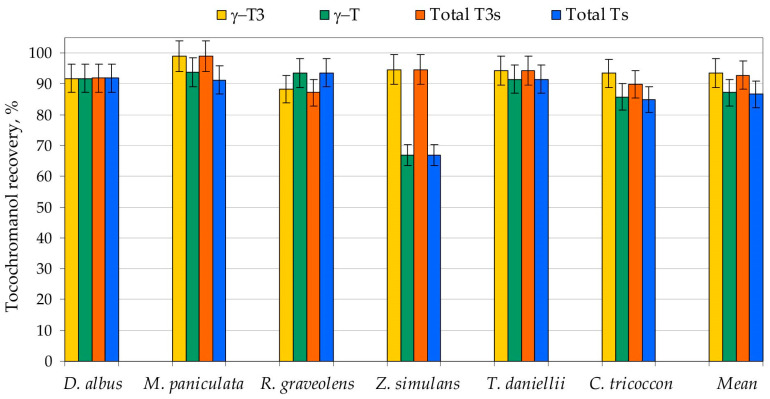
The relative recovery (%) of tocochromanols–total tocopherols (Ts) and tocotrienols (T3s)–from seeds of six Rutaceae species by using the UAEE protocol. Recovery (%) was calculated as an average value for three sample replications and assuming the saponification protocol as 100% recovery of tocochromanols. UAEE, ultrasound-assisted extraction in ethanol.

Saponification is the most common sample preparation protocol for tocopherol and tocotrienol analysis. It has the highest tocochromanol recovery [28], but requires long preparation time, uses nauseating solvents such as hexane, and does not distinguish between free and esterified tocochromanols [29]. This study demonstrates that a substantially simplified UAEE protocol can effectively reduce processing time, labor demands, and solvent toxicity, while still ensuring high tocochromanol recovery and significantly lowering operational costs. The optimised method offers a more sustainable and cost-efficient alternative to conventional procedures without compromising analytical performance for testing a large number of samples. The method has proven suitable for cranberry seed (*Vaccinium macrocarpon*) [30] and grape seed (*Vitis* spp.) [31] tocochromanol analysis and provided recovery similar to a saponification protocol. However, the results should not be compared with saponified sample scores directly and a pilot study with saponification is advisable when investigating new plant material. Esterified tocochromanols can be a minor or major fraction in plant material, their proportion ranging from almost none to almost all tocochromanols. However, that evidence is restricted to a single study, and it considers edible parts of only bell pepper, chili pepper, cucumber, and walnut [32]. Additionally, tocochromanols can be physically bound in the plant material. Therefore, six selected species’ seeds from different genera (*Dictamnus albus*, *Murraya paniculata*, *Ruta graveolens*, *Zanthoxylum simulans*, *Tetradium daniellii*, and *Cneorum tricoccon*) were prepared using both UAEE and saponification protocol for method validation. Recovery differed between tocochromanols and species. Recovery (relative to saponification protocol) of individual tocochromanols is as follows: 88–99%, average 94% for γ-T3 and 67–94%, average 87% for γ-T—detected in all six samples, 84–92%, average 87% for δ-T3 and 59–95%, average 79% for α-T—three samples, 80–96%, average 88% for δ-T—two samples, 80% for β-T3—one sample. α-T3 and β-T were not detected in any of those six samples. Total tocopherols and tocotrienols recovery from seeds of Rutaceae species ranged between 67–94% and 87–99%, and average values 87% and 93%, respectively. Relative recovery (compared to saponification) of individual tocochromanols is depicted in Figure 2, and detailed values are provided in Table S1 (Supplementary Materials).

Slightly higher results obtained for saponified samples in comparison to UAEE can be explained by releasing the free tocochromanols from ester or glucoside derivatives, or non-extractable, physically bonded tocochromanols [29]. Lower extractability of tocopherols than tocotrienols can be an effect not only of higher presence of bound tocopherols, but also their physicochemical properties, especially α-T, resulting in lower recovery [31]. Values and standard error bars reaching over 100% recovery are primarily a consequence of analytical variability inherent to extraction and quantification workflows. Nevertheless, it is also important to recognise that saponification is not a loss-free reference procedure. Degradation of tocochromanols may occur during alkaline hydrolysis, even when pyrogallol is included as a protective antioxidant. Indeed, in grape seeds, saponification has been associated with tocotrienol losses [31]. Due to the low difference between tested protocols, often low concentration of those tocochromanols, challenges in identifying bound tocochromanols, and measurement error, the current study did not attempt to characterise these structures. Both analytical methods exhibited good repeatability, while the UAEE protocol has, on average, a nearly twice lower coefficient of variation for total tocopherols and tocotrienols, emphasizing the usefulness and effectiveness of the application more sustainable protocol for tocochromanol extraction from seeds of Rutaceae plants. Lower repeatability appears associated with low tocochromanol concentration in the plant material (Supplementary Materials). The recovery and repeatability of UAEE protocol is suitable for application in comparative research of Rutaceae family samples with 90–93%, an average of 92%, recovery of total tocochromanols compared to a saponification protocol, demonstrating its suitability for daily sample screening. Since this approach was only tested for six species, comparability and method exchangeability may differ for other species due to potentially different proportion of esterified tocochromanols. However, at present, the literature does not report such cases in seeds, where free tocochromanols predominate [29]. This knowledge gap on bound tocochromanols and their extractability is largely attributable to the fact that comparative extraction studies using both direct and saponification-based protocols are rarely performed in screening studies. Additionally, detecting bound tocochromanols is inherently demanding: it is time-consuming, hindered by the limited availability of appropriate analytical standards, advanced equipment (GC-MS), and further constrained by the generally low concentrations of these compounds in plant tissues [32].

### 2.2. Free Tocochromanol Profile

**Figure 3:**
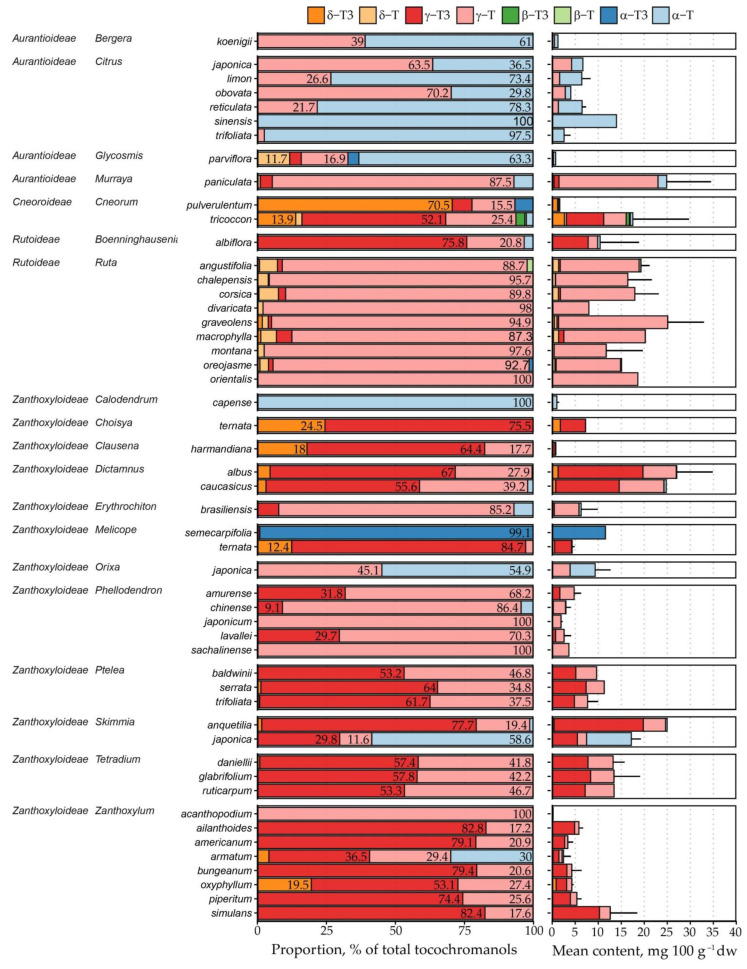
Free tocochromanol mean content and composition in Rutaceae species’ seeds. The data are presented as species mean free tocochromanol contents in a stacked column chart by subfamily and genus.

In total, 135 samples representing 53 species across 19 genera and 4 subfamilies were analysed (Table 1 and Figure 3). The highest tocochromanol content was observed in *R. graveolens* (25.09 ± 7.86, max 42.39 mg 100 g^−1^ dw) and *M. paniculata* (25.98 ± 10.78, max 41.25 mg 100 g^−1^ dw); however, the samples richest in tocochromanols were outliers with about twice the concentration in other biological replicates. The main tocochromanols were γ-T, γ-T3 and α-T. Notably, we observed small quantities of exceptionally rare tocotrienol homologue β-T3 in *C. tricoccon* (0.70 mg 100 g^−1^ dw), together with appreciable levels of α-T3 in *Melicope semecarpifolia* (11.50 mg 100 g^−1^ dw). These distinctive tocochromanol signatures unambiguously set these two species apart from the remaining Rutaceae taxa examined in the present study. Tocotrienol content differed significantly between the species—they could be absent or compose the entirety of free seed tocochromanols. Observed unusually high standard deviation (SD) values relative to their mean tocochromanol contents (variability) for some species can be a result of several factors, such as seed maturity, abiotic factors, genetic background, drying, and storage time. The content of tocochromanols changes during the seed maturity, however; during the last 30 days, the content is nearly fixed, e.g., in Japanese quince (*Chaenomeles japonica*) and grape (*Vitis vinifera*) [25,33]. Botanical gardens harvest seeds at full maturity to preserve viability and germination capacity for the following season. Differences in seed maturity and its effect on the tocochromanol profile and content are therefore negligible. Similarly, low systematic bias (0–2%) can be considered for a strict simplification of seed moisture due to plant material limitation. Abiotic factors have a much greater impact on the tocochromanols profile and content; however, the exact explanation of each of them is complex and ambiguous. For instance, in soybean, it was shown that temperature and water availability affect the profile of tocopherols, while total tocochromanols content was only modestly affected [34]. Many studies unanimously confirm that the genetic background has the most significant impact on the profile and content of tocochromanols in seeds. Tocochromanol content can differ up to two or three times in the seeds of different genotypes belonging to the same species [30,31]. Investigations into conventional thermal drying of *Citrus* seeds (*C.* × *paradisi* × *C. reticulata*, *C. nobilis* × *C. deliciosa*, and *C.* × *limon* ‘*Eureka*’) observed that exposure to high temperatures for prolonged periods of time (60 °C for 24 h) induces only a marginal reduction in tocochromanol content [19]. Given that all seeds examined in the present study were dried at substantially lower temperatures, any drying-related losses of tocochromanols can be assumed to be negligible. Storage of *Brassica napus* seeds stored under suitable conditions for six months showed no detectable tocochromanol degradation [35], while soybean storage under moderate conditions (25 °C; 12% moisture) for twelve months observed a 6–7% tocopherol loss [36]. In summary, the foregoing considerations indicate that the principal drivers of variation in both tocochromanol composition and abundance among the seeds examined are genetic and abiotic factors.

In addition to species provided in Table 1, pomelo (*Citrus maxima*) was analysed, but tocochromanols were essentially absent in the chromatogram. This may be due to malformity of the seeds, since tocopherols and tocotrienols have been reported in pomelo fruit before [16]. In the present study, the pomelo seeds were obtained from a supermarket fruit, and generally they appeared empty. This factor most likely accounts for the differences observed between our results and previously reported data. Moreover, the tocochromanol profiles differ from those obtained for citrus fruit seeds in a previous investigation, where hexane-extracted grapefruit, orange, lemon and mandarin orange seed oil was investigated using the same sample preparation protocol [17] and tocochromanol composition was similar to that observed in pomelo fruits with sample saponification [16]. The results are similar to one study where citrus seeds were investigated [13], but tocotrienol standards were not used. While the difference may in part be explained by a different variety—honey pomelo and golden pomelo tocochromanol profiles differ significantly, as do tocochromanol synthesis patterns during maturation [16].

### 2.3. Free Tocochromanol Composition as Shaped by Phylogeny

There was no apparent single trend between tocochromanols in the Rutaceae family. Within subfamilies, strong association can be observed between γ-T and δ-T3 in Cneoroideae and Rutoideae (ρ = 0.973 and 0.784, respectively), between γ-T and δ-T3 in Aurantioideae, Cneoroideae (ρ = 0.677 and 0.996, respectively), and γ-T and γ-T3 in Aurantioideae, Cneoroideae and Zanthoxyloideae (ρ = 0.878, 0.959, and 0.548, respectively). However, the strength and significance of correlations in Cneoroideae is exaggerated by the low number of representatives. Paired plots, density plots and correlations are provided in Figure S2 (Supplementary Materials).

**Figure 4:**
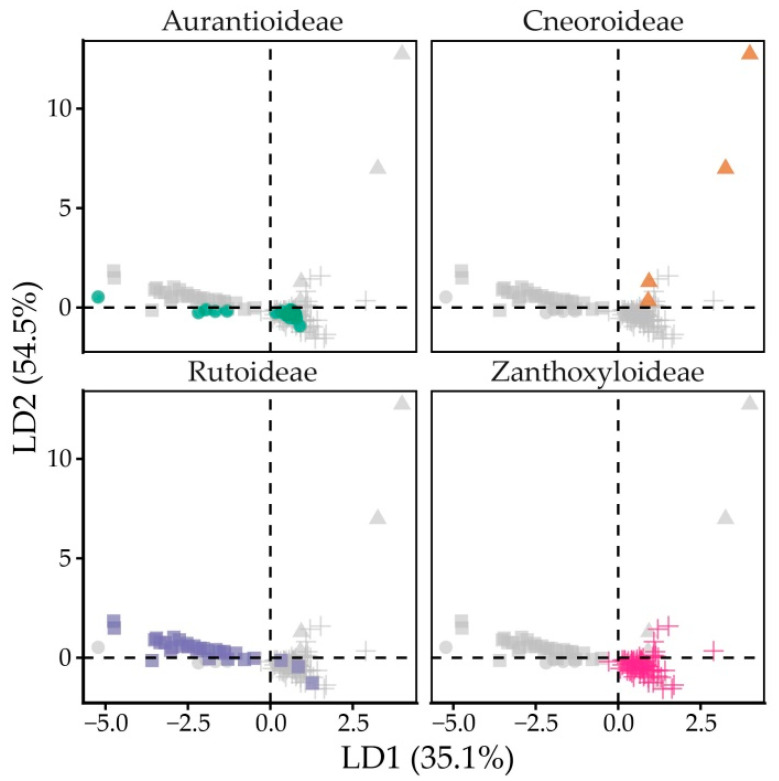
Linear discriminant plot, faceted by subfamily. Colour and shape denote subfamily. Greyed-out datapoints represent the whole dataset.

Tocochromanol composition was subfamily (*p* < 0.001) and genus-dependent (*p* < 0.001) with statistical significance. Following linear discriminant analysis, linear discriminants LD1 and LD2 explain 54.5 and 35.1% of variance between subfamilies, respectively, and identified β-T3 and δ-T3 as the strongest discriminating variables (coefficients > 1). LD1 was positively associated with β-T3 (2.79) and δ-T3 (0.21), and negatively associated with δ-T (−0.83), β-T (−0.87) and γ-T (−0.14). LD2 was positively associated with δ-T3 (5.89), β-T3 (1.25) and δ-T3 (0.49), and negatively associated with β-T (−0.72). Detailed LD loadings are provided in Table S3 (Supplementary Materials). Although LDA identified β-T3 as a major discriminating tocochromanol, it is a minor compound, and mean content was similar between subfamilies with only trace amounts present in a single species (*Cneorum tricoccon*) in variable amounts. The second major discriminating tocochromanol, δ-T3, was not present in Aurantioideae seeds, was present in trace amounts in Rutoideae (0.18 mg 100 g^−1^ dw) and Zanthoxyloideae (0.19 mg 100 g^−1^ dw), but is a consistent minor compound in Cneoroideae (2.28 mg 100 g^−1^ dw). The subfamilies differed in terms of major seed tocochromanols (Table S4, Supplementary Materials)—Cneoroideae and Zanthoxyloideae had higher γ-T3 content (6.10 and 4.90 mg 100 g^−1^ dw, respectively), mean γ-T content was much higher in Rutoideae (16.78 mg 100 g^−1^ dw), compared to Aurantioideae (5.65 mg 100 g^−1^ dw), Cneoroideae (3.73 mg 100 g^−1^ dw) and Zanthoxyloideae (2.89 mg 100 g^−1^ dw). Only Aurantioideae seeds consistently had significant α-T content (3.26 mg 100 g^−1^ dw), while in other subfamilies, the mean was below 1 mg 100 g^−1^ dw. Figure 4 shows subfamily separation along LD1 and LD2, and partial overlap of Zanthoxyloideae and Rutaceae, a cluster made up of the Zanthoxyloideae subfamily and most Aurantioideae (except *Murraya*) and Rutoideae (*Boenninghausenia albiflora* and one *Ruta montana* sample) datapoints, while Cneoroideae is divergent from other subfamilies.

**Figure 5:**
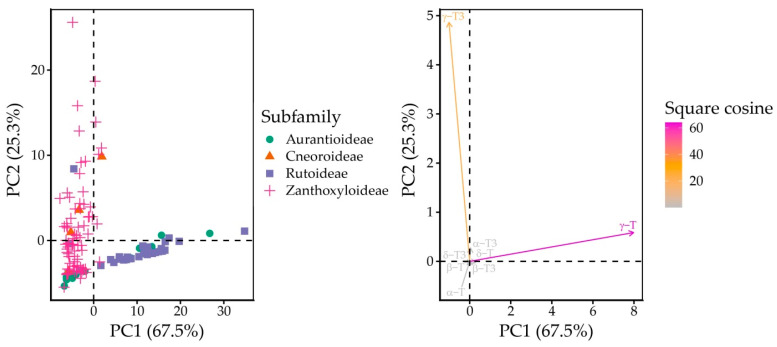
PCA biplot of variables and individual points against PC1 and PC2.

Principal component analysis identified γ-T and γ-T3 as the main discriminating tocochromanols and α-T as a minor discriminating tocochromanol. PC1 and PC2 explain 67.51 and 25.30% of variance, respectively, for a total of 92.82% (Table S5, Supplementary Materials). The standard deviations for PC1 and PC2 were 8.063 and 4.940, respectively. PC1 had a very high loading with γ-T (0.990), while PC2 had a high loading with γ-T3 (0.986). Loading was very low for other tocochromanols (Table S6, Supplementary Materials). Like LDA, PCA indicates similarities between Aurantioideae and Rutoideae, but also classifies Cneoroideae and Zanthoxylum as similar on account of higher γ-T3 content and low or absent γ-T, as shown in Figure 5.

K-means cluster analysis classified the datapoints into two clusters of sizes 103 and 31, explaining 54.8% of variance. Like PCA, the data were not normalised to avoid noise created by minor compounds. Major tocochromanols differed between clusters (Table S6, Supplementary Materials). Cluster 1 had a higher γ-T3 (4.06 mg 100 g^−1^ dw), γ-T (2.71 mg 100 g^−1^ dw), and α-T (1.13 mg 100 g^−1^ dw), while cluster 2 essentially only had high γ-T content (19.44 mg 100 g^−1^ dw), and other tocochromanol mean contents were below 1 mg 100 g^−1^. Although not a major tocochromanol according to PCA, α-T content was also higher in cluster 1. Moreover, total tocochromanol content was lower in cluster 1 than cluster 2, which were further differentiated by high tocopherol and tocotrienol proportion, respectively. Intermediary tocochromanols (γ- chromanol ring structures) were favoured in all groups.

**Figure 6:**
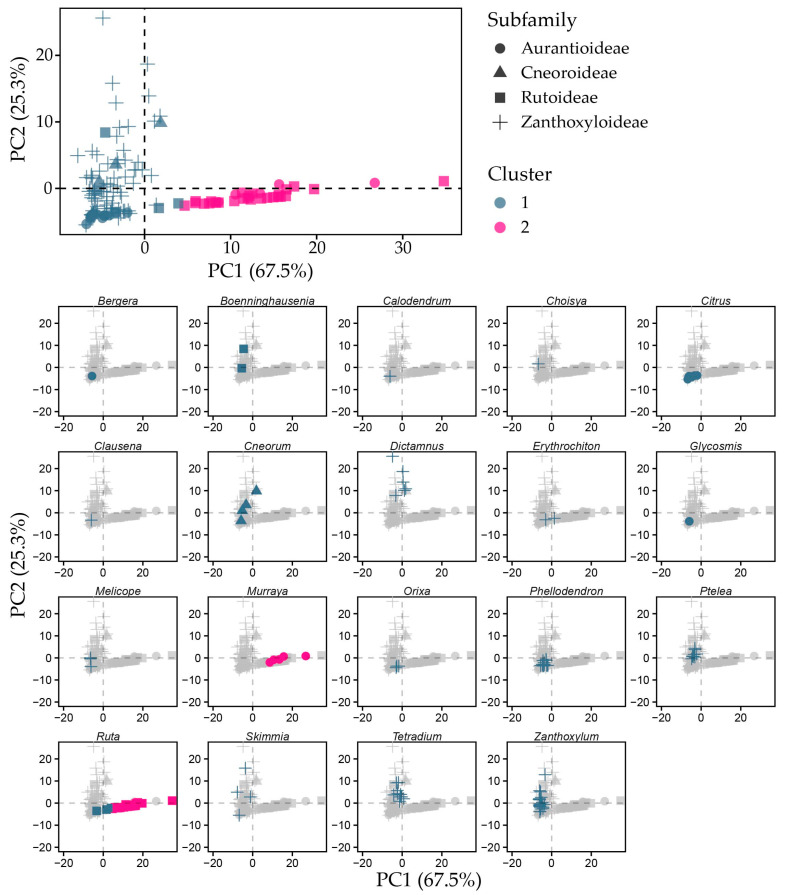
K-means clusters according to tocochromanol composition. All datapoints are represented at top, and separated by genus with species names provided inside the plot, coloured by cluster and shaped by subfamily.

As shown in Figure 6, grouping Rutaceae species by seed tocochromanol composition divides subfamilies between clusters, while most genera are not split between clusters. Cluster 1 was primarily made up of Rutoideae and Aurantioideae datapoints. Zanthoxyloideae and Cneoroideae were placed entirely in cluster 2, as was most of Aurantioideae, while most Rutoideae datapoints were placed in cluster 2.

**Figure 7:**
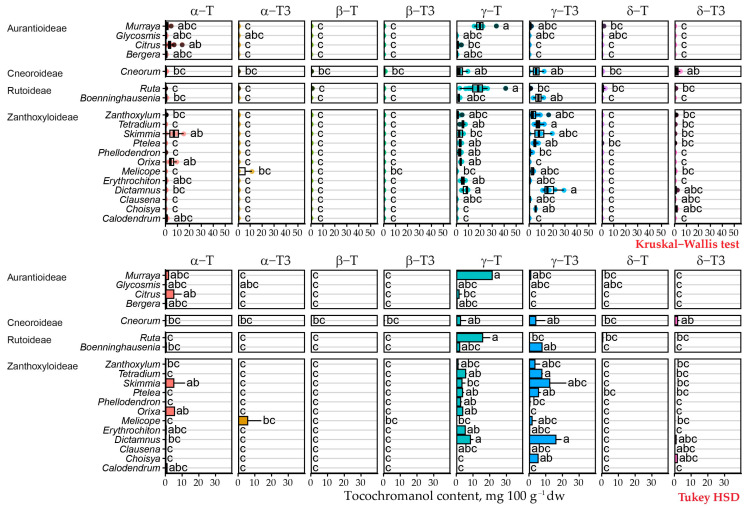
Statistically significant difference between different Rutaceae genera tocochromanol profiles. Data are presented as (**top**) boxplots of the whole dataset with coloured points representing individual samples, black points representing outlier values, and letters denoting statistically similar groups according to the Kruskal–Wallis test, and as (**bottom**) mean tocochromanol content in genus + standard deviation and letters denoting statistically similar groups according to the Tukey HSD test. In both cases, the colour or fill denotes the tocochromanol.

Rutoideae and Aurantioideae species’ datapoints are strewn across one trendline primarily related to PC1, while Zanthoxyloideae species are more spread out and varied across a separate trendline primarily dependent on PC2. Natural variability appears high in several genera: *Boennighausenia*, *Cneorum*, *Dictamnus*, *Murraya*, *Skimmia*, *Tetradium* and *Zanthoxylum*, where datapoints do not form tight clusters of genus or species within the dimension 1 and 2 plot or are placed within different clusters. Others, such as *Calodendrum*, *Erythrochiton*, *Glycosmis*, *Melicope* and *Orixa* clustered close together within species and genus, while *Boennighausenia*, *Cneorum*, *Dictamnus*, *Murraya*, *Ruta, Skimmia*, *Tetradium* and *Zanthoxylum* have longer clusters. *Ruta* is the only genus split between clusters. Most of the genus was in cluster 2, with single datapoints from *R. corsica*, *R. divaricata*, and *R. montana* in cluster 1, which appears to be due to lower γ-T content in these individual samples. PC1 scores are closely correlated to δ-T (0.614) and γ-T (0.997), while dimension 2 scores are correlated with δ-T3 (0.468) and γ-T3 (0.978) with statistical significance (*p* < 0.001). In chemotaxonomic terms, this places genera and species in tocochromanol-low, tocotrienol-rich and tocopherol-rich categories. Correlations between tocochromanols in k-means clusters are depicted in Figure S3 (Supplementary Materials). Statistically significant difference between different Rutaceae genera tocochromanol profiles are shown in Figure 7.

In citrus fruits, species phylogeny within the Aurantioideae subfamily did not seem to cause similar tocochromanol profiles. For example, *C. limon* and *C. sinensis* had quite different tocochromanol composition although their DNA is quite similar, and *C. limon* was instead more similar to *C. reticulata*, though it has been grouped in a different branch of the *Citrus* genus [37,38], while other investigated species did not overlap with existing literature. Species within genera differ significantly in terms of genetic similarity across the Rutaceae family. In certain branches of the Rutaceae family, genetic similarity is loosely reflected in the tocochromanol profile—*Zanthoxylum*, *Tetradium* and *Phellodendron*, less so *Dictamnus albus* (all in the Zanthoxyloideae subfamily), are quite genetically similar [39], and visually so are their tocochromanol profiles, but *Murraya*, *Citrus*, *Clausena*, and *Glycosmis* species are relatively genetically similar [39], but their tocochromanol profiles differ significantly from each other. Varied tocochromanol composition in the *Zanthoxylum* genus cannot be explained entirely by genetic differences—*Z. acanthopodium* and *Z. armatum* are very close phylogenetically [40], but their tocochromanol compostition is unlike observed for the other investigated species. This may be caused by sampling issues, as some fruit was too small and tough to separate seeds from the rind, which affected tocochromanol composition. However, discrepancies may also be caused as a result of different seed development.

Phylogeny-shaped lipid antioxidant profiles have been observed in other plant families as well. Phylogeny affects legume tocochromanol and carotenoid profiles in Fabaceae species [41], and consistent tocochromanol profiles are observed in certain monocot families, such as Arecaceae or palm family [42], and eudicot families, such as Apiaceae [43,44,45]. In addition, tocochromanol content can be affected by environmental factors. For example, carotenoid and tocochromanol contents differ between legume species with natural ranges in temperate and tropical climates [41], and in palm oil the tocochromanol content differs between populations [46].

## 3. Materials and Methods

### 3.1. Reagents

Potassium hydroxide, pyrogallol, sodium chloride (reagent grade), *n*-hexane, methanol, ethyl acetate, ethanol (HPLC grade) were obtained from Sigma-Aldrich (Steinheim, Germany). The 96.2% ethanol was received from Kalsnavas Elevators (Jaunkalsnava, Latvia). The eight tocochromanol standards, α, β, γ, and δ homologue of tocopherols and tocotrienols (>95%, HPLC) were purchased from LGC Standards (Teddington, Middlesex, UK) and Merck (Darmstadt, Germany).

### 3.2. Plant Material

In total, 135 samples belonging to 53 species, 18 genera and 4 subfamilies of Rutaceae family were analysed. Some common citrus fruit (lemon, orange, mandarin orange and pomelo) seeds were manually removed from fruits purchased in local stores. Some of the seed samples were collected from local wild plants (*Ruta graveolens* and *Phellodendron amurense*), and certain spices (*Zanthoxylum*) were purchased from local stores. Other seed samples were obtained from botanical gardens across the world, mainly Eurasia, Austria (AT), Belgium (BE), Czech Republic (CZ), Estonia (EE), France (FR), Georgia (GE), Germany (DE), Hungary (HUN), Italy (IT), Latvia (LV), Poland (PL), Portugal (PT), Romania (RO), Russia (RU), Slovakia (SK), Slovenia (SI), Spain (ES), Taiwan (TW), Ukraine (UA), and the United States (US). Seeds were collected in full maturity, air-dried (20–30 °C), and stored in the dark to give germination ability in the next season. Seeds were sent via mail in paper or plastic bags, as part of seed exchange programs between botanical gardens. The obtained seeds were catalogued as they were received and cleaned from other plant part residues, e.g., fruit flesh, if required and possible. As more than 90% of the seed material was acquired through formal seed exchange programs among botanical gardens, and considering the small amount of seed available per accession together with the exceptionally large number of samples processed during the project (>10,000), the deposition of voucher specimens was not feasible. Consequently, voucher or specimen numbers are not available. To ensure transparency, the provenance of each analysed sample is documented in the Supplementary Materials as the donating institution.

The Rutaceae family was one of over a hundred families investigated as part of this project. The species verification of provided plant material (seeds) was performed by the staff of the donor botanical garden which shared with their genotype resources. To reduce the impact of such factors as risk of misidentified species, crossbreeding, environmental factors, and others, origin (botanic garden) diversification was prioritised alongside species diversity. Synonymic species were checked using online databases, such as wikispecies.com (classification into subfamilies) and worldfloraonline.com (synonymic species), using the consensus or most recent reference available. Original species names, and number of replications for each species, and the full list of botanical gardens that supported this project are provided in Supplementary Materials. Seeds were obtained and analysed between 2019 and 2024. Upon receipt, samples were analysed within ~6–12 months after the collection of seeds. The seeds were stored in paper and plastic bags, away from direct light, under room-temperature conditions (21 ± 3 °C). Seed moisture can be estimated at 10 ± 3%. To remove residual moisture before grinding, the seeds were frozen at −80 °C for 1–3 h, freeze-dried using a FreeZone freeze-dry system (Labconco, Kansas City, MO, USA) at a temperature of −51 ± 1 °C and <0.01 mbar for 24–48 h, depending on the size and number of seeds. Lyophilised seeds contained 3–7% moisture. Due to generally limited seed mass, an average moisture content of value 5% was used as default/constant for tocochromanol calculation for all samples. Dry seeds (0.1–1 g) were powdered using an MM 400 mixer mill (Retsch, Haan, Germany) and tocochromanols were extracted within the same day using an ultrasound-assisted extraction and 96.2% ethanol (UAEE), as described below in Section 3.3.2. (all samples), and Section 3.3.1. for six randomly selected samples (recovery study).

### 3.3. Tocochromanols Extraction

#### 3.3.1. Saponification

The saponification protocol of powdered seed samples (0.05–0.10 g) was performed in the presence of 2% pyrogallol (an antioxidant) in ethanol (*w*/*v*) and 60% aqueous potassium hydroxide (*w*/*v*), incubated in a water bath at 80 °C for 25 min. After saponification, tocochromanols were extracted three times using *n*-hexane:ethyl acetate solution (9:1, *v*/*v*). The details of the saponification step and subsequent tocochromanol extraction are reported earlier [30]. The organic solvent was evaporated, dissolved in 1 mL of ethanol, transferred to 2 mL glass vials and analysed immediately by a reversed-phase liquid-chromatography system with fluorescence detection (RPLC-FLD).

#### 3.3.2. Ultrasound-Assisted Extraction in Ethanol (UAEE)

The greener method was adopted from a developed protocol for extraction of tocochromanols in cranberry seeds [30]. Briefly, the powdered seeds (0.05–0.10 g) were placed in a 15 mL tube and supplemented with 96.2% ethanol (*v*/*v*) (5 mL), mixed (1 min) at 3500 rpm using vortex and treated by ultrasound of nominal ultrasonic power 160 W and ultrasound frequency 35 kHz using Sonorex RK 510 H ultrasonic bath (Bandelin Electronic, Berlin, Germany) at 60 °C for 15 min. Immediately after completion of the ultrasonic step, the samples were mixed (1 min) as before, centrifuged at 11,000× *g* at 21 °C for 5 min, and transferred directly to a 2 mL glass vial and analysed in a RPLC-FLD system.

#### 3.3.3. Method Validation

Since the extraction of tocopherols and tocotrienols from all tested seeds using UAEE differs from most studies investigating tocochromanol content in plant material, the results were compared with standard saponification protocol. Recovery (%) tests of tocopherols and tocotrienols from the seeds of six selected species from different genera (*Dictamnus albus*, *Murraya paniculata*, *Ruta graveolens*, *Zanthoxylum simulans*, *Tetradium daniellii*, and *Cneorum ricoccon*) (6 × 3 UAEE vs. 6 × 3 saponification) were performed. Measurement repeatability (%) for both extraction protocols was evaluated. Repeatability (coefficient of variation) was calculated based on the independent determinations of a sample by analyzing three replicates on the same day. Error of measurement (standard deviation) was calculated based on the independent determinations of a sample by analyzing three replicates on the same day.

### 3.4. Tocochromanol Determination by Reversed-Phase Liquid-Chromatography with Fluorescent Detection (RPLC-FLD)

Determination of four tocopherols and four tocotrienols was completed according to a reported method using authentic standards and calculated using calibration curves produced earlier [28]. Separation was performed on a Luna PFP column (3 µm, 150 × 4.6 mm) (Phenomenex, Torrance, CA, USA) using 93% methanol (*v*/*v*) as mobile phase with 1 mL/min flow rate and 40 °C temperature of column-oven. Measurements were completed on a LC 10 series (Shimadzu, Kyoto, Japan) system equipped with a RF-10AXL fluorescence detector using the following detection parameters of excitation and emission λ_ex_ = 295 nm and λ_em_ = 330 nm, respectively. Details of method validation are provided in the Table S1 and Figure S1 (Supplementary Materials).

### 3.5. Statistical Analysis

The results of all the performed experiments of different species seed samples are presented as means ± standard deviation (*n* = 1–9). Statistical analyses were performed without data transformation, and, to retain as much natural variability as possible in the dataset, potential outlier values were included. Multivariate analysis of variance (MANOVA) with Tukey HSD post-hoc and the Kruskal–Wallis test were used to determine statistically significant differences between subfamilies and genera, and linear discriminant analysis was used to determine statistically similar subfamilies. The main differentiating tocochromanols were identified using principal component analysis (PCA), and samples were grouped based on tocochromanol composition using non-hierarchal k-means cluster analysis. Base and opensource R (R 4.3.2) packages stats, agricolae, MASS, factoextra and dplyr were used for data analysis, and opensource packages tidyr, dplyr, ggplot2, ggpubr, gghighlight, ggrepel, ggtext, patchwork, and scales were used for data reshaping and visualization using RStudio 2025.09.2+418 “Cucumberleaf Sunflower” Release (12f6d5e22720bd78dbd926bb344efe12d0dce83d, 20 October 2025) for Windows.

## 4. Conclusions

It is clear that a significant proportion of Rutaceae species’ seed free tocochromanols are tocotrienol-dominated, and that presumed tocotrienol scarcity in eudicot plants is a result of underreporting rather than fact. Tocochromanol synthesis preference in the Rutaceae family is strongly phylogeny-determined and seed tocochromanol composition is generally similar between closely related plants, as are the relationships between different individual tocochromanol contents. Species are most distinctly differentiated by their γ-T and γ-T3 content. Species in the Rutoideae and Aurantioideae subfamily have tocopherol-dominated seed tocochromanol profiles. In Aurantioideae, the main seed tocochromanol is α-T, while in Rutoideae, γ-T constituted a majority of total free tocochromanols. Cneoroideae (*Cneorum* sp.) and Zanthoxyloideae seed free tocochromanols are generally tocotrienol-dominated, but their proportion is less consistent than in Aurantoideae and Rutoideae.

UAEE had good repeatability and slightly lower recovery than the saponification protocol, and UAEE can be considered a reliable tocochromanol extraction method for Rutaceae family seeds. It can be recommended for routine monitoring, but should be validated for any new plant material, since it only allows for extracting free tocochromanols, and information on the proportion of esterified and otherwise bound tocochromanols is very limited at this time.

## Figures and Tables

**Table 1:** Free tocochromanol content in seeds of Rutaceae species (mg 100 g^−1^ dw).

Genus	Species	δ-T3	γ-T3	δ-T	γ-T	α-T	Total
Aurantioideae subfamily							
*Bergera*	*koenigii* (*n* = 1)	ND	ND	ND	0.50	0.80	1.26
*Citrus*	*japonica* (*n* = 1)	ND	ND	ND	4.20	2.40	6.67
	*limon* (*n* = 3)	ND	ND	ND	1.6 ± 0.10	4.80 ± 2.00	6.42 ± 1.91
	*obovata* (*n* = 1)	ND	ND	ND	2.80	1.20	4.04
	*reticulata* (*n* = 3)	ND	ND	ND	1.33 ± 1.03	5.13 ± 1.79	6.50 ± 0.84
	*sinensis* (*n* = 1)	ND	ND	ND	ND	14.00	13.96
	*trifoliata* (*n* = 5)	ND	ND	ND	0.02 ± 0.04	2.58 ± 1.41	2.59 ± 1.4
*Murraya*	*paniculata* (*n* = 5)	ND	1.10 ± 0.66	0.40 ± 0.79	21.48 ± 7.27	1.88 ± 1.76	24.87 ± 9.64
*Glycosmis*	*parviflora* (*n* = 2)	ND	ND	0.10 ± 0	0.10 ± 0	0.50 ± 0.14	0.76 ± 0.11
Cneoroideae subfamily							
*Cneorum*	*pulverulentum* (*n* = 1)	1.20	0.10	ND	0.30	ND	1.64
	*tricoccon* (*n* = 3)	2.63 ± 2.16	8.10 ± 4.06	0.43 ± 0.42	4.87 ± 4.24	0.60 ± 0.56	17.52 ± 12.21
Rutoideae subfamily							
*Boenninghausenia*	*albiflora* (*n* = 2)	ND	7.80 ± 6.22	ND	2.05 ± 1.48	0.55 ± 0.78	10.42 ± 8.47
*Ruta*	*angustifolia* (*n* = 3)	0.13 ± 0.15	0.37 ± 0.47	1.23 ± 0.25	17.13 ± 2.11	0.03 ± 0.06	19.29 ± 1.87
	*chalepensis* (*n* = 5)	ND	0.04 ± 0.09	0.70 ± 0.45	15.70 ± 4.97	ND	16.43 ± 5.23
	*corsica* (*n* = 5)	0.10 ± 0.12	0.42 ± 0.13	1.24 ± 0.34	16.18 ± 4.87	ND	17.95 ± 5.17
	*divaricata* (*n* = 1)	ND	ND	0.20	7.80	ND	7.97
	*graveolens* (*n* = 9)	0.46 ± 0.26	0.33 ± 0.52	0.58 ± 1.03	23.70 ± 7.50	ND	25.08 ± 7.86
	*macrophylla* (*n* = 1)	0.30	1.10	1.10	17.70	0.10	20.25
	*montana* (*n* = 3)	ND	ND	0.43 ± 0.75	11.3 ± 7.44	ND	11.73 ± 7.95
	*oreojasme* (*n* = 1)	0.10	0.30	0.50	14.00	ND	15.07
	*orientalis* (*n* = 1)	ND	ND	ND	18.60	ND	18.60
Zanthoxyloideae subfamily							
*Calodendrum*	*capense* (*n* = 2)	ND	ND	ND	ND	1.00 ± 0.57	1.01 ± 0.51
*Clausena*	*harmandiana* (*n* = 1)	0.10	0.50	ND	0.10	ND	0.77
*Dictamnus*	*albus* (*n* = 5)	1.26 ± 0.69	18.44 ± 7.22	ND	7.24 ± 2.42	0.12 ± 0.18	27.07 ± 7.8
	*caucasicus* (*n* = 1)	0.80	13.80	ND	9.70	0.50	24.78
*Melicope*	*semecarpifolia* (*n* = 1)	ND	0.10	ND	ND	ND	11.57
	*ternata* (*n* = 2)	0.55 ± 0.07	3.65 ± 0.35	ND	0.15 ± 0.07	ND	4.33 ± 0.54
*Orixa*	*japonica* (*n* = 3)	ND	ND	ND	3.87 ± 0.64	5.47 ± 3.48	9.34 ± 3.35
*Phellodendron*	*amurense* (*n* = 5)	ND	1.66 ± 1.24	ND	3.12 ± 0.85	ND	4.77 ± 1.48
	*chinense* (*n* = 3)	ND	0.23 ± 0.40	ND	2.63 ± 1.40	0.10 ± 0.17	3.00 ± 1.08
	*japonicum* (*n* = 2)	ND	ND	ND	1.95 ± 0.35	ND	1.91 ± 0.36
	*lavallei* (*n* = 3)	ND	0.73 ± 0.59	ND	1.83 ± 1.07	ND	2.57 ± 1.54
	*sachalinense* (*n* = 1)	ND	ND	ND	3.60	ND	3.64
*Skimmia*	*anquetilia* (*n* = 1)	0.40	19.40	ND	4.80	0.30	24.96
	*japonica* (*n* = 3)	ND	5.47 ± 5.03	ND	2.00 ± 3.46	9.73 ± 5.26	17.20 ± 2.04
*Tetradium*	*daniellii* (*n* = 7)	0.09 ± 0.23	7.70 ± 2.58	ND	5.46 ± 1.56	ND	13.23 ± 2.50
	*glabrifolium* (*n* = 2)	ND	8.35 ± 6.01	ND	5.05 ± 0.35	ND	13.41 ± 5.69
	*ruticarpum* (*n* = 1)	ND	7.20	ND	6.30	ND	13.43
*Zanthoxylum*	*acanthopodium* (*n* = 1)	ND	ND	ND	0.20	ND	0.20
	*ailanthoides* (*n* = 3)	ND	4.90 ± 1.48	ND	0.90 ± 0.52	ND	5.80 ± 0.90
	*americanum* (*n* = 2)	ND	2.70 ± 1.13	ND	0.65 ± 0.07	ND	3.39 ± 1.18
	*armatum* (*n* = 6)	0.18 ± 0.40	1.28 ± 1.21	ND	0.68 ± 0.61	0.33 ± 0.28	2.46 ± 1.61
	*bungeanum* (*n* = 2)	ND	3.20 ± 0.99	ND	1.05 ± 1.06	ND	4.25 ± 2.14
	*oxyphyllum* (*n* = 2)	0.90 ± 0.85	2.30 ± 0.71	ND	1.10 ± 1.13	ND	4.26 ± 0.38
	*piperitum* (*n* = 3)	ND	3.93 ± 1.21	ND	1.40 ± 1.21	ND	5.36 ± 1.01
	*simulans* (*n* = 4)	ND	10.28 ± 4.29	ND	2.33 ± 1.66	ND	12.59 ± 5.87
*Choisya*	*ternata* (*n* = 1)	1.80	5.50	ND	ND	ND	7.24
*Erythrochiton*	*brasiliensis* (*n* = 2)	ND	0.45 ± 0.07	ND	5.35 ± 3.18	0.45 ± 0.35	6.29 ± 3.62
*Ptelea*	*baldwinii* (*n* = 1)	ND	5.10	ND	4.50	ND	9.64
	*serrata* (*n* = 1)	0.10	7.20	ND	3.90	ND	11.29
	*trifoliata* (*n* = 6)	0.05 ± 0.12	4.78 ± 1.59	ND	2.88 ± 0.77	ND	7.70 ± 2.25

Data are presented as means within species ± standard deviation, except in cases where only one seed sample was obtained and the quantified free tocochromanol content is provided. T3, tocotrienol; T, tocopherol; ND, not detected. In addition, β-T3, α-T3 and β-T were very minor compounds and only present in trace amounts in a few species: β-T3 in *C. tricoccon* (0.70 ± 0.67 mg 100 g^−1^ dw), α-T3 in *Cneorum pulverulentum* (0.10 mg 100 g^−1^ dw), *Ruta oreojasme* (0.20 mg 100 g^−1^ dw) and *M. semecarpifolia* (11.50 mg 100 g^−1^ dw), and β-T in *C. tricoccon* (0.17 ± 0.30 mg 100 g^−1^ dw) and *Ruta angustifolia* (0.37 ± 0.25 mg 100 g^−1^ dw). *Citrus maxima* seeds were analysed, but tocochromanols were not detected.

## Data Availability

Data are contained within the article and Supplementary Materials.

## Supplementary Materials

The following supporting information can be downloaded at: https://www.mdpi.com/article/10.3390/plants15030455/s1, Figure S1: Chromatogram of the RP-HPLC-FLD separation of four tocopherol (T) and four tocotrienol (T3) homologue standards; Figure S2: Trends and Spearman rank correlation between individual tocochromanols in four analysed Rutaceae subfamilies; Figure S3: Individual tocochromanol, PC1 and PC2 score Spearman rank correlation in identified k-means clusters; Table S1: Linearity, limit of detection (LOD), limits of quantification (LOQ), and retention time (RT) of the RP-HPLC-FLD method for tocochromanols determination; Table S2: Repeatability (%) for the determination of tocopherols and tocotrienols in the seeds of Rutaceae family.

## Abbreviations

RP-HPLC, reverse-phase liquid chromatography; GGPP, geranylgeranyl pyrophosphate; PPP, phytyl pyrophosphate; MEP, methyl erythritol pathway; HGA, homogentisate; T, tocopherol; T3, tocotrienol.
